# Lychee-like TiO_2_@Fe_2_O_3_ Core-Shell Nanostructures with Improved Lithium Storage Properties as Anode Materials for Lithium-Ion Batteries

**DOI:** 10.3390/ma16051945

**Published:** 2023-02-27

**Authors:** Yuan Chen, Feihong Liu, Yufei Zhao, Mengdie Ding, Juan Wang, Xuan Zheng, Huihu Wang, Marie-Christine Record, Pascal Boulet

**Affiliations:** 1 Hubei Provincial Key Laboratory of Green Materials for Light Industry, Collaborative Innovation Center of Green Light-Weight Materials and Processing, and School of Materials and Chemical Engineering, Hubei University of Technology, Wuhan 430068, China; 2 New Materials and Green Manufacturing Talent Introduction and Innovation Demonstration Base, Wuhan 430068, China; 3 Hubei Longzhong Laboratory, Xiangyang 441000, China; 4 Aix-Marseille University, Faculty of Sciences, IM2NP, CEDEX 20, 13397 Marseille, France; 5 Aix-Marseille University, Faculty of Sciences, Madirel, CEDEX 20, 13397 Marseille, France

**Keywords:** lithium-ion batteries, anode materials, TiO_2_@Fe_2_O_3_ microspheres, electrochemical properties, first-principles calculations

## Abstract

In this study, lychee-like TiO_2_@Fe_2_O_3_ microspheres with a core-shell structure have been prepared by coating Fe_2_O_3_ on the surface of TiO_2_ mesoporous microspheres using the homogeneous precipitation method. The structural and micromorphological characterization of TiO_2_@Fe_2_O_3_ microspheres has been carried out using XRD, FE-SEM, and Raman, and the results show that hematite Fe_2_O_3_ particles (7.05% of the total mass) are uniformly coated on the surface of anatase TiO_2_ microspheres, and the specific surface area of this material is 14.72 m^2^ g^−1^. The electrochemical performance test results show that after 200 cycles at 0.2 C current density, the specific capacity of TiO_2_@Fe_2_O_3_ anode material increases by 219.3% compared with anatase TiO_2_, reaching 591.5 mAh g^−1^; after 500 cycles at 2 C current density, the discharge specific capacity of TiO_2_@Fe_2_O_3_ reaches 273.1 mAh g^−1^, and its discharge specific capacity, cycle stability, and multiplicity performance are superior to those of commercial graphite. In comparison with anatase TiO_2_ and hematite Fe_2_O_3_, TiO_2_@Fe_2_O_3_ has higher conductivity and lithium-ion diffusion rate, thereby enhancing its rate performance. The electron density of states (DOS) of TiO_2_@Fe_2_O_3_ shows its metallic nature by DFT calculations, revealing the essential reason for the high electronic conductivity of TiO_2_@Fe_2_O_3_. This study presents a novel strategy for identifying suitable anode materials for commercial lithium-ion batteries.

## 1. Introduction

With the increase in energy consumption and environmental issues, lithium-ion batteries have attracted considerable interest as a form of energy storage technology [1,2,3]. Due to its low specific capacity and poor cycle performance at high current densities, it is increasingly challenging for commercial graphite to match the needs of lithium-ion batteries [4,5]. As a result, new commercial anode materials are required [6]. Due to their theoretical capacity, high power density, and abundance of resources, transition metal oxides have become a research hotspot for lithium-ion battery anode materials [7]. Nevertheless, the majority of transition metal oxides (including Fe_2_O_3_ [8], Fe_3_O_4_ [9], MnO_2_ [10], Mn_2_O_3_ [11], Mn_3_O_4_ [12], MnO [13], CuO [14], NiO [15], and Co_3_O_4_ [16]) used as anode materials for lithium-ion batteries typically experience significant volume expansion effects during the lithium insertion/extraction process. As an anode material for lithium-ion batteries, TiO_2_ possesses high insertion/extraction lithium-ion potential and a stable insertion/extraction lithium-ion structure in addition to high coulombic efficiency, cheap cost, stable chemical characteristics, and environmental friendliness [17,18]. However, the low electronic conductivity of TiO_2_ anode materials, as well as the low diffusion coefficient of lithium-ion within them, is insufficient for commercial applications.

Currently, TiO_2_ anode material optimization measures are primarily focused on two aspects: nanosizing and material compounding [4]. The nanoscale TiO_2_ anode materials can increase the contact area between the anode materials and the electrolyte, reduce the lithium-ion transport channel within the materials, and provide additional lithium-ion storage sites. Yao et al. [19] manufactured hollow spherical TiO_2_ anode materials, Zhao et al. [20] constructed interconnected TiO_2_ nanofiber anode materials, and Lu et al. [21] produced nanowire TiO_2_ anode materials. Compounding TiO_2_ with other materials can also effectively enhance the performance of anode materials [22,23,24,25]. Guan et al. [26] coated TiO_2_ with TiOF_2_, Wang et al. [27] covered a layer of MgCo_2_O_4_ on the surface of TiO_2_, and Li et al. [28] compounded TiO_2_ with C/FeS_2_.

Fe_2_O_3_, as a transition metal oxide, has the advantages of low cost, high theoretical capacity, abundant resources, and low power density, and therefore has attracted the interest of researchers [29,30]. The literature indicates that Fe_2_O_3_ and TiO_2_, two anode materials, have a synergistic effect [30,31], i.e., the composite of structurally stable TiO_2_ and Fe_2_O_3_ with a high theoretical capacity can effectively raise the specific capacity of the anode materials. In addition, these materials are inexpensive, abundant in resources, environmentally friendly and pollution-free, and appropriate for large-scale applications. The majority of current studies on the composite of these two materials concentrate on using Fe_2_O_3_ as a substrate and utilizing TiO_2_ as a stable coating layer to address the problem of high-volume expansion of Fe_2_O_3_ [4,25,31]. However, less research has been conducted on the issue of the poor specific capacity of TiO_2_ when combined with Fe_2_O_3_ on a TiO_2_ substrate.

In this study, the lychee-like core-shell structure TiO_2_@Fe_2_O_3_ microspheres were prepared by coating a layer of Fe_2_O_3_ particles on the surface of homemade TiO_2_ mesoporous microspheres using the homogeneous precipitation method. In order to investigate the connection between the structure of the materials and their electrochemical performance, we first conducted the phase analysis, micromorphology characterization, and BET test on TiO_2_ mesoporous microspheres and TiO_2_@Fe_2_O_3_ microspheres; subsequently, we tested and compared the electrochemical performance of these two materials as anode materials for lithium-ion batteries and discussed the differences in the electrochemical performance of the materials before and after coating. To further explore the synergistic effect of TiO_2_ and Fe_2_O_3_, calculations using density functional theory were made for TiO_2_, Fe_2_O_3_, and TiO_2_@Fe_2_O_3_. The results indicated that the composite of TiO_2_ and Fe_2_O_3_ changed the electron density of states of the material, which led to a significant increase in electrical conductivity. This research offers a novel strategy for identifying suitable anode materials for commercial lithium-ion batteries.

## 2. Materials and Methods

### 2.1. Materials

The raw materials consisted of urea (CH_4_N_2_O, Sinopharm Chemical Reagent Co., Shanghai, China, AR), anhydrous ethanol (C_2_H_5_OH, Sinopharm Chemical Reagent Co., Shanghai, China, AR), FeCl_3_·6H_2_O (Aladdin Reagent Co., Shanghai China, ACS), Fe_2_O_3_ (Heng Xing Reagent Co., Tianjin, China, AR), commercial graphite (C, Tianjin Battery Co., Tianjin, China, AR) and tetrabutyl titanate (TBOT, Shanghai Macklin Biochemical Co., Shanghai, China, AR). Solutions were prepared with deionized water (Molecular Lab water ultra-purifier, Shanghai, China).

The precursor TiO_2_ microspheres used in this study were homemade using a low-temperature modified Stöber method, which was previously reported by our group [32]. In this method, aqueous KCl and tetrabutyl titanate were added to ethanol and reacted at a low temperature of −10 °C for 5 h. The precursor was then collected by centrifugation, washed three times with alcohol and once with water, and subsequently freeze-dried, resulting in amorphous TiO_2_ microspheres.

### 2.2. Synthesis of Core-Shell Structure TiO_2_@Fe_2_O_3_ Microspheres

**Figure 1:**
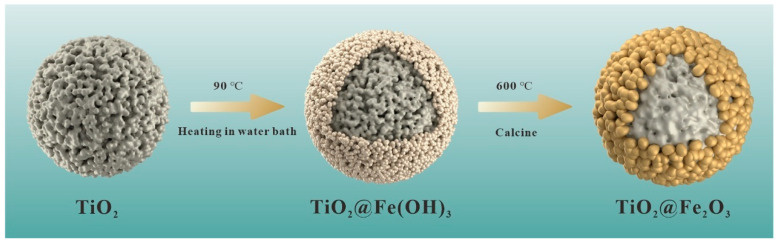
Preparation schematic of TiO_2_@Fe_2_O_3_ microspheres.

Figure 1 illustrates the preparation method for TiO_2_@Fe_2_O_3_ microspheres with the core-shell structure. The above-mentioned TiO_2_ microspheres (0.3 g) were first ultrasonically dispersed in 100 mL of deionized water, followed by the addition of FeCl_3_*·*6H_2_O (0.5 g) and 0.5 g urea under magnetic stirring for 10 min. The above suspensions were further heated in an 80 °C water bath with magnetic stirring for 8 h to obtain intermediate microspheres according to the following reaction Equations (1) to (3):

(NH_2_)_2_CO(aq) + 3H_2_O→2NH_4_OH(aq) + CO_2_(g)↑ (1)

NH_4_OH(aq)→NH_4_^+^(aq) + OH^−^ (2)

[Fe(H_2_O)_6_]^3+^(aq) + 3OH^−^→Fe(OH)_3_(s)↓ + 6H_2_O (3)

The precipitate was then collected by centrifugation, washed three times with deionized water, and dried for 12 h at 50 °C. The sample was then calcined at 600 °C for 4 h. The morphology of the sample can be altered by varying the amount of FeCl_3_·6H_2_O added. The equation corresponding to this reaction is shown in Equation (4).

2Fe(OH)_3_→Fe_2_O_3_ + 3H_2_O (4)

### 2.3. Structure Analysis and Characterization

The structural properties of the products were characterized by X-ray diffraction (XRD) using an X-ray diffractometer (PANalytical, Empyrean, Almelo, Netherlands) with Cu Kα radiation (*λ* = 0.154 nm) and a 2*θ* scan range of 20~90° at a rate of 2°/min. Raman spectra were recorded on an XploRA PLUS spectrometer (HORIBA, Lille, France) using a 532 nm wavelength laser source.

The morphologies and elemental ratio of the samples were determined by a field emission scanning electron microscope (FE-SEM, SU8010, Hitachi, Tokyo, Japan) operating at 5 kV with an energy dispersive X-ray spectrometer (EDS, X-MaxN, OXFORD, Oxford, UK).

The N_2_ adsorption/desorption isotherms of porous products were measured using a surface area and pore size analyzer (BET, ASAP 2020 PLUS HD88, Micromeritics, Norcross, GA, USA). The specific surface area was calculated with the Brunauer—Emmett—Teller (BET) model, and the pore size distribution was evaluated using the Barrett—Joyner—Halenda (BJH) method.

### 2.4. Electrochemical Tests

The anode materials, cotinine black (Shanghai Macklin Biochemical Co., Shanghai, China, AR), and the polyvinylidene fluoride (PVDF, Shanghai Macklin Biochemical Co., Shanghai, China, AR) were weighed according to the mass ratio of 8:1:1, thoroughly mixed, and the mixed sample was then placed in a beaker with N-Methylpyrrolidone (NMP, Shanghai Aladdin Biochemical Co., Shanghai, China, AR) to be magnetically stirred for 7 h. The slurry was coated on 20 μm thick copper foil, fully dried at 80 °C, and finally cut into 15 mm diameter discs. The solution of 1 M LiPF_6_ was dissolved in ethylene carbonate (EC) and dimethyl carbonate (DMC) as electrolyte (EC:DMC = 1:1 vol%). Polypropylene film (Celgard 2500, Celgard Inc., Charlotte, NC, USA) was used as a separator, lithium foil was used as counter and reference electrode, and CR2032 half-cells were assembled in a glove box in an argon atmosphere. The mass loading of the electrodes was around 1.0–1.4 mg cm^−2^. A Land CT2001A battery test system was used to measure the charge/discharge cycling performance and rate capability of the anode materials in the voltage range of 0.01~3.0 V (vs. Li/Li^+^). Cyclic voltammetry (CV) analysis of the anode materials was performed using an electrochemical workstation (CS350M, Corrtest Instrument Corp., Ltd., Wuhan, China) with a voltage window of 0.01~3.5 V and a scan rate of 0.1 mV·s^−1^. Electrochemical impedance spectra (EIS) were tested on the same instrument in the frequency range from 1 × 10^6^ to 0.01 Hz.

### 2.5. Computational Details

The density functional theory (DFT) with the pseudopotential plane-wave (PP-PW) approach, as implemented in the VASP program package [33,34], was used to compute the total energy and density of states of TiO_2_, Fe_2_O_3,_ and TiO_2_@Fe_2_O_3_. Specifically, we employed the generalized gradient approximation (GGA) [35,36] functionals as proposed by Perdew and Wang (PW91) [37]. The cutoff energy of the plane-wave basis sets was set at 500 eV, and self-consistent electronic steps and structure relaxation were carried out until the energy and force thresholds of 1 × 10^−6^ eV/atom and 0.01 eV/Å/atom, respectively, were reached. TiO_2_ and Fe_2_O_3_ computations were conducted using 4 × 4 × 4 Monkhorst-Pack k-point meshes. By contrast, a Monkhorst-Pack k-point mesh of 4 × 2 × 2 was used for TiO_2_@Fe_2_O_3_ due to the large number of atoms (i.e., 299 atoms) contained in the supercell of TiO_2_@Fe_2_O_3_. Every simulation run required two processes to calculate the density of states: (1) optimizing the crystal structure to obtain the lowest possible total energy, and (2) performing self-consistent simulations using the optimized structure.

## 3. Results and Discussion

### 3.1. Structural Characterization

**Figure 2:**
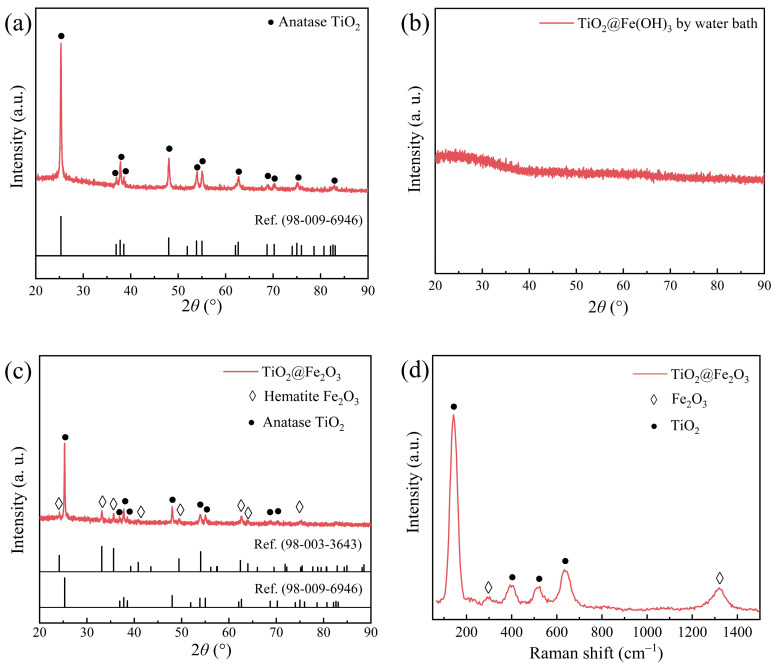
XRD patterns of (**a**) TiO_2_ after 600 °C calcination, (**b**) TiO_2_@Fe(OH)_3_ prepared by water bath, (**c**) TiO_2_@Fe_2_O_3_ microspheres, (**d**) Raman spectra of TiO_2_@Fe_2_O_3_ microspheres.

Previously, we reported that the TiO_2_ mesoporous microspheres produced in our laboratory at low temperature were amorphous [32]. Figure 2a shows the X-ray diffraction pattern of the TiO_2_ mesoporous microspheres after calcination at 600 °C. All the diffraction peaks in the pattern correspond to the anatase TiO_2_ (ref. code: 98-009-6946) in the tetragonal crystal system, indicating that the structure of the TiO_2_ mesoporous microspheres after calcination at 600 °C is the anatase phase.

Figure 2b shows the X-ray diffraction pattern of TiO_2_@Fe(OH)_3_ microspheres generated by water bath reaction prior to calcination. There are no discernible diffraction peaks in the pattern, indicating that both TiO_2_ and hydroxide precipitates of Fe in TiO_2_@Fe(OH)_3_ microspheres are in an amorphous state prior to calcination. Figure 2c shows the X-ray diffraction pattern of TiO_2_@Fe(OH)_3_ microspheres after calcination at 600 °C for 4 h. The results show that all the diffraction peaks of the sample correspond to those of hematite α-Fe_2_O_3_ (ref. code: 98-003-3643) in the hexagonal crystal system and anatase TiO_2_ (ref. code: 98-009-6946) in the tetragonal crystal system. This indicates that the Fe(OH)_3_ precipitation is transformed to α-Fe_2_O_3_ through calcination, following Equation (4).

Figure 2d shows the Raman spectrum pattern of calcined TiO_2_@Fe_2_O_3_ microspheres. Raman characteristic peaks of the sample at 142, 394, 525, and 634 cm^−1^ correspond to the E_g(1)_, B_1g_, A_1g_, and E_g(2)_ vibrational modes of anatase TiO_2_ [38], while Raman characteristic peaks of the sample at 302 and 1318 cm^−1^ correspond to the E_g(1)_ + E_g(2)_, E_g(3)_ vibrational modes of hematite Fe_2_O_3_ [31]. No characteristic peaks corresponding to the vibrational modes of other phases are detected, providing additional evidence that the TiO_2_@Fe_2_O_3_ microspheres are composed of anatase TiO_2_ and hematite Fe_2_O_3_.

The above structural characterization demonstrates that after calcination, TiO_2_ in TiO_2_@Fe_2_O_3_ microspheres prepared by water bath reaction transforms from the early amorphous state to the anatase phase, and the hydroxide precipitation of Fe transforms to α-Fe_2_O_3_ of hematite phase, with no other impurities being produced.

### 3.2. Morphological Characterization

**Figure 3:**
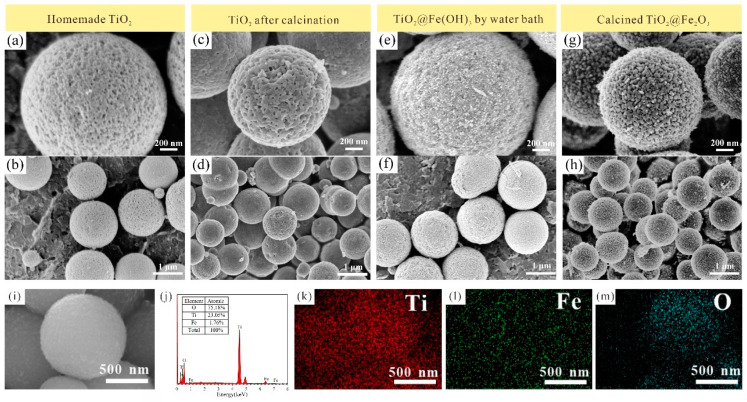
FE-SEM images of the (**a**,**b**) homemade TiO_2_, (**c**,**d**) TiO_2_ after calcination at 600 °C, (**e**,**f**) TiO_2_@Fe(OH)_3_ prepared by water bath, and (**g**,**h**) calcined TiO_2_@Fe_2_O_3_ microspheres; (**i**,**j**) the EDS spectrum and (**k**–**m**) mapping images of TiO_2_@Fe_2_O_3_ microspheres. The magnification of (**a**,**c**,**e**,**g**,**i**) is 60 k, and that of (**b**,**d**,**f**,**h**) is 20 k.

Figure 3a,b display the FE-SEM images of the TiO_2_ mesoporous microspheres fabricated in our laboratory by the low-temperature modified Stöber method, from which it can be observed (1) that the surface of the microspheres is rough, (2) that there is a large number of mesopores, (3) the average diameter of the microspheres is 1~2 μm, and (4) the monodispersity of the microspheres is good, which is consistent with our previous work [32]. Figure 3c,d show the FE-SEM images of the TiO_2_ mesoporous microspheres after calcination at 600 °C. After calcination, the surface of the TiO_2_ mesoporous microspheres becomes smooth and some mesopores are closed, which may have been caused by the decrease in the surface area of the microspheres during the phase transition of TiO_2_, hence reducing the overall surface energy of the system. The particle size of the microspheres reduced after the calcination step. This may be due to the presence of a large number of micropores inside the uncalcined microspheres. After the calcination, because of the occurrence of crystallization, some of the stomata of this microsphere became closed, decreasing the microsphere volume. Figure 3e,f show the FE-SEM images of TiO_2_@Fe(OH)_3_ microspheres prepared by water bath reaction, from which it can be observed that the TiO_2_@Fe(OH)_3_ microspheres are uniform in size and well dispersed; compared with Figure 3a, the surface of the microspheres in Figure 3e is rougher and wrapped with a coating layer composed of fine particles, which may be the hydroxide precipitation of Fe according to the previous inference. Figure 3g,h reveal the FE-SEM images of the calcined TiO_2_@Fe_2_O_3_ microspheres. Compared with Figure 3e, the particles covered on the surface of the microsphere sample in Figure 3g are larger, and these particles are interconnected and interlaced with one another. The surface of the sample is lychee-like, and it is inferred from the results in Figure 2d that the particulate matter covered on the surface should be α-Fe_2_O_3_; Figure 3h shows that the monodispersity of the microspheres remains good after the calcination. The above microscopic morphological characterization indicates that after the calcination, some of the micropores on the surface of TiO_2_ microspheres inside the sample are closed, and the surface coating layer is transformed from fine hydroxide precipitation of Fe to larger Fe_2_O_3_ particles.

To determine the elementary composition of the calcined TiO_2_@Fe_2_O_3_ microspheres, the FE-SEM image of the analysis area and the associated EDS spectrum are depicted in Figure 3i,j, respectively. It is well indicated that the TiO_2_@Fe_2_O_3_ microspheres consist of Ti, Fe, and O elements, and the Ti/Fe atomic ratio is 1.75:23.05. The Fe element is significantly less than the Ti element, implying the thin thickness of the obtained Fe_2_O_3_ coating layer. To have a clear insight into the distribution of elements in the TiO_2_@Fe_2_O_3_ microspheres, elemental mappings based on the analysis area are derived, as shown in Figure 3k–m. Perspicuously, the profiles of three-element mapping show a uniform distribution of Ti, Fe, and O elements, which further indicates a better compound between Fe_2_O_3_ and TiO_2_. The theoretical specific capacity *C* of the TiO_2_@Fe_2_O_3_ microspheres is calculated from the Fe/Ti atomic ratio, molar masses of TiO_2_ and Fe_2_O_3_, theoretical specific capacity of TiO_2_ and Fe_2_O_3_ and Equation (5) and is 382.38 mAh g^−1^ [39,40].

*C*(TiO_2_@Fe_2_O_3_) = *C*(TiO_2_) × wt%(TiO_2_) + *C*(Fe_2_O_3_) × wt%(Fe_2_O_3_) (5) = 335 × 92.95% + 1007 × 7.05% = 382.38 mAh g^−1^

### 3.3. BET Analysis

**Figure 4:**
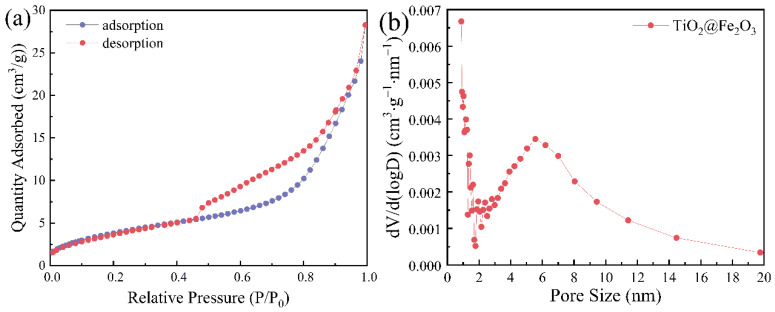
(**a**) N_2_ adsorption (purple)—desorption (red) isotherms and (**b**) pore size distribution curves of TiO_2_@Fe_2_O_3_ microspheres.

As shown in Figure 4a, the nitrogen adsorption—desorption isotherms of the TiO_2_@Fe_2_O_3_ microspheres exhibit the type IV nitrogen adsorption branch with a type-H3 hysteresis loop, confirming the presence of mesopores in the TiO_2_@Fe_2_O_3_ microspheres [7]. The Brunauer—Emmett—Teller (BET) surface area is calculated to be 14.72 m^2^/g. Figure 4b shows the corresponding Barrett—Joyner—Halenda (BJH) pore-size distribution of the pores in the sample, and it can be seen that the pore size in the sample is mainly distributed from 1 to 10 nm, and the average pore volume is calculated to be 0.0437 cm^3^/g. The results show that after calcination, the pores in TiO_2_@Fe_2_O_3_ microspheres are mainly micropores and mesopores, which is consistent with the SEM observation.

### 3.4. Electrochemical Performance

**Figure 5:**
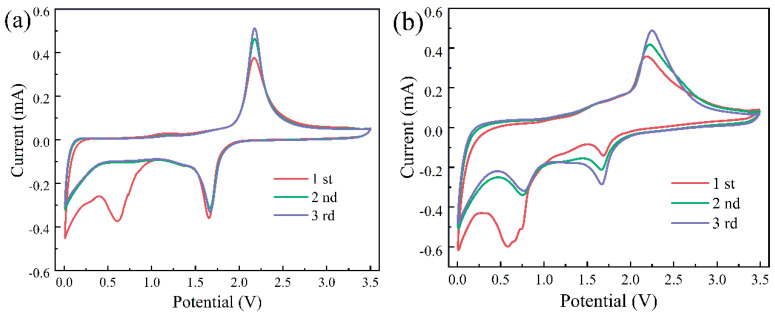
CV curves of (**a**) anatase TiO_2_ and (**b**) TiO_2_@Fe_2_O_3_ microspheres in the potential window of 0.01~3.5 V with a scanning rate of 0.1 mV·s^−1^.

To investigate the influence of the Fe_2_O_3_ coating layer on the electrochemical performance of TiO_2_, we fabricated half-cells with the calcined TiO_2_ (anatase) and TiO_2_@Fe_2_O_3_ as anode materials (working electrode), tested and compared their electrochemical performance. To compare the lithiation reactions of TiO_2_, Fe_2_O_3_, and TiO_2_@Fe_2_O_3_ while observing whether there are other side reactions of homemade TiO_2_, we chose a large test voltage range from 0.01 to 3.5 V. Figure 5a,b show the cyclic voltammetry curves of the above two microspheres for the initial three cycles at a scan rate of 0.1 mV/s. As shown in Figure 5a, for calcined TiO_2_, a distinct reduction peak first appears at 1.7 V during the first discharge, which together with the oxidation peak at 2.2 V during the charging process corresponds to the characteristic peaks of the lithium-ion insertion/extraction reactions in anatase TiO_2_ [8], as shown in Equation (6).

TiO_2_ + *x*Li^+^ + *x*e^−^ ↔ Li*_x_*TiO_2_ (6)

Subsequently, a significant reduction peak appears at 0.6 V and disappears in subsequent cycles, which may be due to the insertion of Li^+^ into the irreversible site and the generation of the solid electrolyte interphase (SEI) layer [38,39]. In the two subsequent cycles, there is a tendency for the CV curves to overlap, indicating a gradual stabilization of the lithium-ion insertion/extraction reaction in the TiO_2_ anode material.

As shown in Figure 5b, for the TiO_2_@Fe_2_O_3_ microsphere anode material, the reduction peak first appears at 1.7 V during the first discharge, and this peak similarly corresponds to the characteristic peak of the Li^+^ insertion/extraction reaction in anatase TiO_2_ together with the oxidation peak at 2.2 V during the charging process. Subsequently, a continuous reduction peak at 0.4~1.0 V corresponds to the multistep reduction of Fe_2_O_3_ to Fe^0^ according to Equations (7) and (8), which shifts to a reduction peak at 0.7 V during the subsequent cycles [25,30]. Presumably, the disappeared reduction peak corresponds to the insertion of Li^+^ into the irreversible site and the generation of the SEI layer [31] (Figure S1 shows the CV curve of hematite Fe_2_O_3_, in which these peaks also appear, further proving that these peaks belong to Fe_2_O_3_). This reduction peak at 0.7 V together with the broad oxidation peak at 1.5~2.0 V during the charging process corresponds to the characteristic peaks of the Li^+^ insertion/extraction reactions in α-Fe_2_O_3_ as shown in Equation (9).

Fe_2_O_3_ + 2Li^+^ + 2e^−^ → Li_2_(Fe_2_O_3_) (7)

Li_2_(Fe_2_O_3_) + 4Li^+^ + 4e^−^ → 2Fe^0^ + 3Li_2_O (8)

Fe_2_O_3_ + 6Li ↔ 2Fe + 3Li_2_O (9)

Therefore, the total Li^+^ insertion/extraction reactions of TiO_2_@Fe_2_O_3_ microsphere anode material are shown in Equations (6) and (9). In the two subsequent cycles, the CV curves tend to overlap, demonstrating that the Li^+^ insertion/extraction reactions in TiO_2_@Fe_2_O_3_ gradually stabilize.

**Figure 6:**
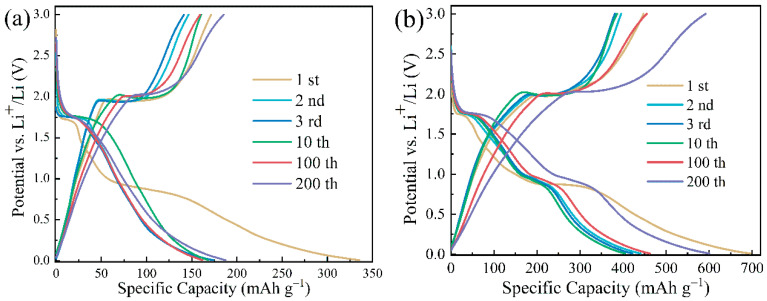
Galvanostatic discharge/charge curves of the 1st, 2nd, 3rd, 10th, 100th, and 200th cycles for (**a**) anatase TiO_2_ and (**b**) TiO_2_@Fe_2_O_3_ microspheres in the voltage range of 0.01~3.0 V at 0.2 C current density.

Figure 6a depicts the charge/discharge curves of the anode material calcined anatase TiO_2_ at a current density of 0.2 C. During the first discharge, a distinct plateau appears at 1.75 V. This plateau together with the one appearing at 2.0 V during the charging process corresponds to the insertion/extraction process of lithium-ions in the interstitial octahedral sites of anatase TiO_2_, with the chemical reaction shown in Equation (6). Subsequently, a slow ramp appears between 0.9~0.7 V and disappears in the subsequent cycles, which may be due to the generation of the SEI layer and the insertion of Li^+^ into the irreversible site [38,39]. This result is in accordance with the analysis of Figure 5a. In the first charge/discharge cycle, the initial discharge- and charge-specific capacities of the battery are 335.2 and 178.6 mAh g^−1^, respectively, corresponding to the initial coulombic efficiency (ratio of charge capacity to discharge capacity) of only 53.3%; the large capacity loss may be attributed to the generation of the SEI layer and the insertion of Li^+^ into the irreversible site [38,39].

Figure 6b shows the charge/discharge curves of the TiO_2_@Fe_2_O_3_ anode material at a current density of 0.2 C. Similarly, the plateau that appears at 1.75 V during the first discharge along with the plateau at 2.0 V during the charging process corresponds to the insertion/extraction process of lithium-ions in the interstitial octahedral sites of anatase TiO_2_. Subsequently, a distinct plateau occurs around 0.8 V, which evolves into a section of slope at 1.0~0.7 V during the following cycles. This slope, together with the slope at 1.5~2.0 V during charging, corresponds to the insertion/extraction process of lithium-ions in α-Fe_2_O_3_ as shown in Equation (9), and this finding is consistent with the analysis of Figure 5b. The initial discharge- and charge-specific capacities of the TiO_2_@Fe_2_O_3_ anode material are 698.5 and 476.5 mAh g^−1^, respectively, and the reversible capacity (the portion of capacity that can participate in both charging and discharging within a single cycle, i.e., the minimum of the discharge-specific capacity and the charge-specific capacity) is 476.5 mAh g^−1^, which is larger than the theoretical specific capacity 382.38 mAh g^−1^ of TiO_2_@Fe_2_O_3_ previously obtained by calculation (see Equation (5)), which may be mainly attributed to the synergistic effect of TiO_2_ and Fe_2_O_3_ [30,31] and the additional lithium storage sites provided by the larger specific surface area of the material (the surface of the material is more likely to form point defects, thus providing more lithium storage sites and increasing the specific capacity of the material) [40]. The initial coulombic efficiency of the material is 68.2%, and the lost capacity can still be attributed to the generation of the SEI layer and the insertion of Li^+^ into the irreversible site.

**Figure 7:**
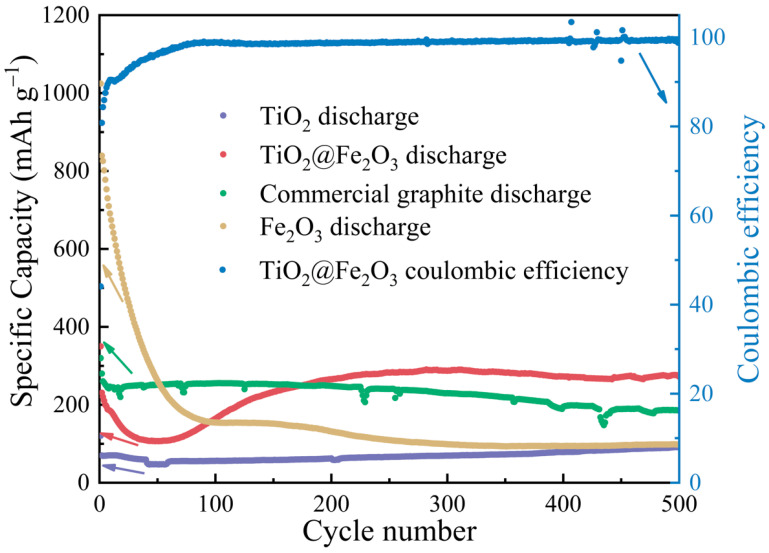
Cycling performances of anatase TiO_2_, TiO_2_@Fe_2_O_3_, hematite Fe_2_O_3_, commercial graphite and coulombic efficiency of TiO_2_@Fe_2_O_3_ in the voltage range of 0.01~3.0 V at 2 C current density.

After 2, 3, 10, 100, and 200 cycles, the reversible capacities of TiO_2_@Fe_2_O_3_ anode material are 395.6, 381.0, 386.1, 455.2, and 591.9 mAh g^−1^, which are much larger than those of anatase TiO_2_ anode material (146.5, 141.4, 160.1, 159.1, and 185.4 mAh g^−1^). It is interesting to note that the reversible capacity of the TiO_2_@Fe_2_O_3_ material at the 200th cycle is increased by 219.3% compared with TiO_2_ and is much higher than its own theoretical specific capacity of 335.2 mAh g^−1^. According to the results of the previous EDS analysis, the mass of coated Fe_2_O_3_ in TiO_2_@Fe_2_O_3_ microspheres only accounts for 7.05% of the total mass, and the coating of such a small amount of Fe_2_O_3_ brings a significant increase in the reversible capacity of the material, implying a good synergistic effect of TiO_2_ and Fe_2_O_3_ as anode materials. The reversible capacity of both anatase TiO_2_ and TiO_2_@Fe_2_O_3_ microspheres is enhanced after a large number of cycles, which is a phenomenon commonly found in transition metal oxides. This may be due to the structural changes induced in the anode material when lithium-ions are continuously inserted/extracted, i.e., further activation of the material [7,40,41]. It is noteworthy that the reversible capacity of TiO_2_@Fe_2_O_3_ microsphere anode material at the 200th cycle increased by 49.62% compared with the 2nd cycle, which is significantly higher than that of TiO_2_ by 26.81%. The reason may be that the TiO_2_@Fe_2_O_3_ generates new interfaces after the coating of Fe_2_O_3_, which reduces the activation energy of this material. With the continuous insertion/extraction of lithium-ions, the structure of the active material is more likely to change, resulting in the formation of more new effective lithium storage sites within the material during the cycling process, which leads to an increase in specific capacity. This finding is also supported by the results in Figure 7 below.

To further exhibit the cycling performance as well as the rate capabilities of the materials, we prepared half-cells with anatase TiO_2_ microspheres, TiO_2_@Fe_2_O_3_ microspheres, commercial graphite, and α-Fe_2_O_3_ calcined at 600 °C as the anode material (working electrode), and performed cyclic charge/discharge tests at a current density of 2 C; their discharge specific capacities are shown as the purple, red, green, and brown curves in Figure 7, respectively. The discharge-specific capacities of anatase TiO_2_ and TiO_2_@Fe_2_O_3_ microspheres after 500 cycles are 91.6 and 273.9 mAh g^−1^ at a current density of 2 C, respectively, indicating that the TiO_2_@Fe_2_O_3_ microsphere anode material with Fe_2_O_3_ coating exhibits a significant increase in the specific capacity after a large number of cycles, and this result is consistent with the findings demonstrated in Figure 6b. In addition, after 500 cycles, the coulombic efficiency of the TiO_2_@Fe_2_O_3_ microsphere reaches 99.4%, indicating that the material has good reversibility of charge and discharge [42]. The discharge-specific capacity of the commercial graphite anode stabilizes from the 10th cycle until the 132nd cycle when it begins to decrease gradually, and the discharge-specific capacity is only 167.1 mAh g^−1^ after 500 cycles, which indicates that the TiO_2_@Fe_2_O_3_ microsphere anode material has superior cycling consistency and specific capacity compared with commercial graphite. The discharge-specific capacity of the hematite Fe_2_O_3_ anode material has been decaying with cycling and is only 98.1 mAh g^−1^ after 500 cycles, which indicates that employing TiO_2_ as the core of the TiO_2_@Fe_2_O_3_ anode material can significantly improve the cycling consistency of this material (the nanoscale structure of Fe_2_O_3_ on the surface of the microsphere also plays an important role). The above results demonstrate that in the TiO_2_@Fe_2_O_3_ microsphere anode material, TiO_2_ as the inner core maintains the structural stability of the material when Li-ions are inserted/extracted, while Fe_2_O_3_ as the outer shell can effectively enhance the specific capacity of the material. We are trying to explain the essential reasons for the synergistic effect of the two materials through computer simulations, which are in progress.

**Figure 8:**
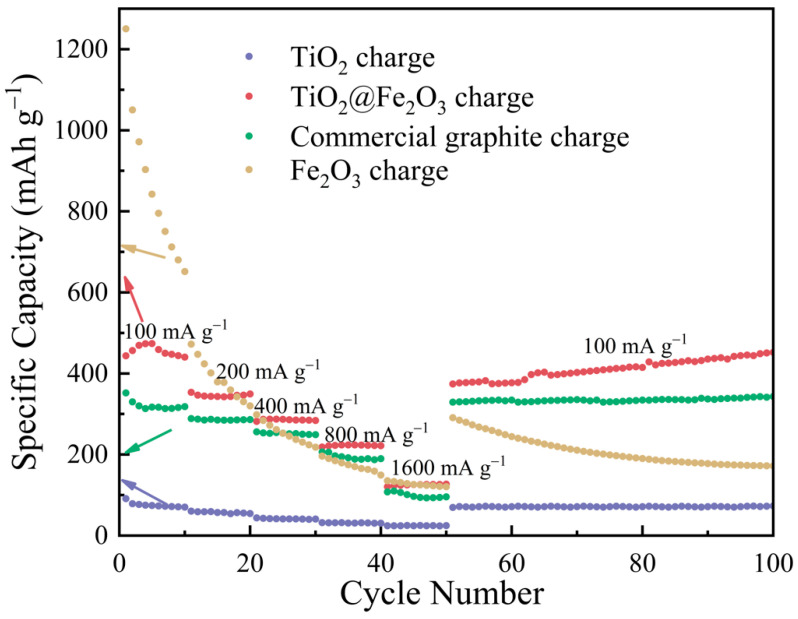
Rate capability tests of anatase TiO_2_, TiO_2_@Fe_2_O_3_, hematite Fe_2_O_3,_ and commercial graphite at rates of 100, 200, 400, 800, 1600, and 100 mA g^−1^ in sequence.

Figure 8 exhibits the rate performance of anatase TiO_2_ microspheres, TiO_2_@Fe_2_O_3_ microspheres, commercial graphite, and the calcined α-Fe_2_O_3_ at 600 °C. At current densities of 100, 200, 400, 800, and 1600 mA g^−1^, the reversible capacities of the anatase TiO_2_ anode materials are 73.8, 57, 41.3, 31, and 24.7 mAh g^−1^, respectively; the reversible capacities of the TiO_2_@Fe_2_O_3_ microsphere anode materials are 394.4, 339.9, 288.2, 220.1, and 126.1 mAh g^−1^, respectively; the reversible capacities of the commercial graphite are 317.0, 284.9, 251.9, 190.6, and 95.7 mAh g^−1^; and the reversible capacities of αFe_2_O_3_ anode materials are 818.8, 378.6, 255.9, 172.5, and 125.6 mAh g^−1^, respectively. The reversible capacities of TiO_2_@Fe_2_O_3_ microsphere anode materials at different current densities are significantly larger than those of TiO_2_ and commercial graphite anode materials, indicating that TiO_2_@Fe_2_O_3_ microsphere anode material has the potential for the application. For the calcined α-Fe_2_O_3_ anode material, the specific capacity decays at different current densities due to the absence of a solid TiO_2_ core to support the structure, and this finding is consistent with the results presented in Figure 7. At the end of the test at the current density of 1600 mA g^−1^, the four materials are then cycled at a current density of 100 mA g^−1^. From the results shown in Figure 8, it can be found that the specific capacities of the three materials—anatase TiO_2_ microspheres, TiO_2_@Fe_2_O_3_ microspheres, and commercial graphite—are not significantly different from those measured at 100 mA g^−1^ at the beginning of the rate performance test, suggesting that the charge-discharge cycles at high current density do not significantly damage the structural integrity of the three materials [43]. The above results show that the rate performance of the TiO_2_@Fe_2_O_3_ microsphere anode material is significantly improved after the coating of Fe_2_O_3_ compared with that of anatase TiO_2_, and we assume that this is because the Fe_2_O_3_ coating layer may improve the electronic conductivity of the material and enhance the lithium-ion diffusion rate in the material.

**Figure 9:**
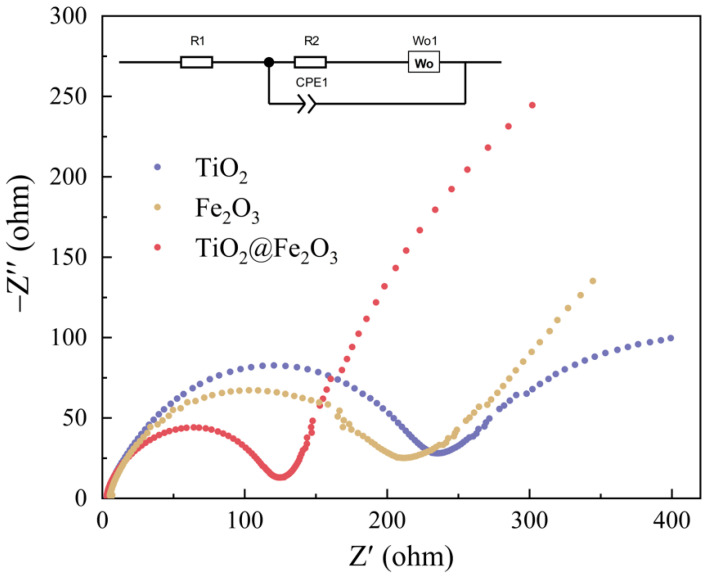
Nyquist plots of anatase TiO_2_, TiO_2_@Fe_2_O_3,_ and hematite Fe_2_O_3_ in the frequency range from 0.01 to 1 × 10^6^ Hz. The inset is the corresponding equivalent circuit.

To further investigate the influence of the Fe_2_O_3_ coating layer on the electronic conductivity of the anode materials, we conducted EIS tests on anatase TiO_2_, TiO_2_@Fe_2_O_3_ microsphere, and hematite Fe_2_O_3,_ respectively, and the results are shown in Figure 9. The Nyquist plots of all three materials consist of a semicircle in the high- and medium-frequency region and a slant line in the low-frequency region. The half circle in the high- and medium-frequency region reflects the impedance of the material, which includes the internal resistance of the cell and the charge transfer impedance, the smaller the radius of the half circle, the smaller the impedance; the slope of the slant line in the low-frequency region reflects the lithium-ion diffusion rate in the anode material, and the greater the slope of the slant line, the faster the diffusion rate of Li-ions [44]. Based on the lithium-ion insertion/extraction mechanism in the anode material, the EIS profile can be fitted based on an equivalent circuit (inset in Figure 9). R1 is the ohmic resistance (total resistance of the electrolyte, separator, and electrical contacts), R2 is the charge transfer resistance, Wo1 is the Warburg impedance of lithium-ion diffusion into the active materials, and CPE1 is the constant phase-angle element, which involves double layer capacitance [45]. The fitted results are shown in Table 1. The charge transfer impedances of anatase TiO_2_ microspheres, TiO_2_@Fe_2_O_3_ microspheres, and α-Fe_2_O_3_ anode materials are 238.35, 119.81, and 202.17 Ω, respectively, and these impedances reflect the conductivity of the three materials, among which, the conductivity of TiO_2_@Fe_2_O_3_ microsphere anode material is significantly higher than that of the other two materials and the results of DFT simulations later in the paper also confirm this finding. For the low-frequency region, the slope of the slant line of TiO_2_@Fe_2_O_3_ microsphere anode material is significantly larger than that of TiO_2_ as well as Fe_2_O_3_, and the slope of the slant line of Fe_2_O_3_ is larger than that of TiO_2_. This indicates that the lithium-ion diffusion rate is the highest in TiO_2_@Fe_2_O_3_ microsphere anode material, which is mainly attributed to the higher lithium-ion diffusion rate of Fe_2_O_3_ (1 × 10^−14^ to 1 × 10^−11^ cm^2^ s^−1^) [46] relative to TiO_2_ (1 × 10^−15^ to 1 × 10^−9^ cm^2^ s^−1^) [7] and the unique porous and core-shell structure of TiO_2_@Fe_2_O_3_ microspheres that shorten the diffusion distance of lithium-ions. The above results reveal that the electronic conductivity of TiO_2_@Fe_2_O_3_ microsphere anode material and the diffusion rate of lithium-ions inside it are significantly improved relative to anatase TiO_2_. It has been reported in the literature [25,47] that the increase in the electronic conductivity of the material and the diffusion rate of lithium-ions inside it can enhance the rate properties of the material, which is consistent with the results demonstrated in Figure 8.

### 3.5. First-Principles Calculations

**Figure 10:**
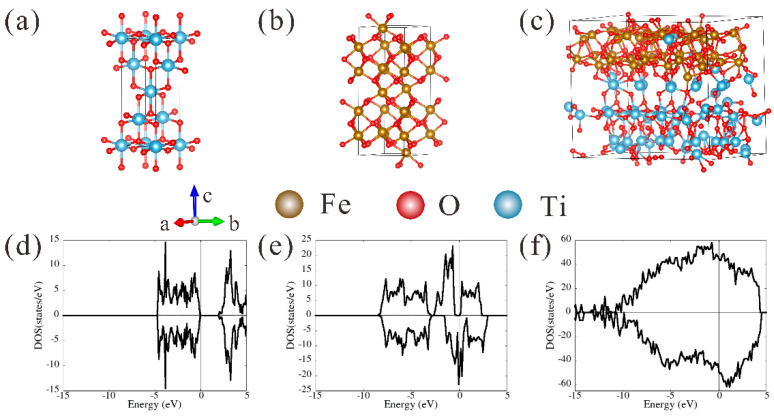
Optimized structures of (**a**) anatase TiO_2_, (**b**) hematite Fe_2_O_3,_ and (**c**) TiO_2_@Fe_2_O_3_; corresponding electron density of states (DOS) of (**d**) anatase TiO_2_, (**e**) hematite Fe_2_O_3,_ and (**f**) TiO_2_@Fe_2_O_3_.

Figure 9 demonstrates that the electronic conductivity of TiO_2_@Fe_2_O_3_ microsphere anode material is significantly greater than that of TiO_2_ and Fe_2_O_3_. To explain this finding and investigate further the synergistic effect of TiO_2_ and Fe_2_O_3_, we used density functional theory to calculate the electron density of states (DOS) of anatase TiO_2_, hematite Fe_2_O_3_, and TiO_2_@Fe_2_O_3_. Figure 10a depicts the crystal structure of anatase TiO_2_, which belongs to the tetragonal system and has structurally optimized lattice constants of *a* = *b* = 3.803 Å and *c* = 9.748 Å. Figure 10d illustrates the DOS map of anatase TiO_2_ with a forbidden bandwidth of 2.14 eV near the Fermi level, indicating that anatase TiO_2_ is a semiconductor with poor conductivity. As shown in Figure 10b, the crystal structure of hematite Fe_2_O_3_ is derived from its XRD test results, which belongs to the hexagonal system with *a* = *b* = 5.035 Å, *c* = 13.763 Å, *α* = *β* = 90°, *γ* = 120°. There is a band gap around 0.4 eV for the majority spin, and no band gap for the minority spin, suggesting the hematite Fe_2_O_3_ is a half-metal with slightly higher electrical conductivity than anatase TiO_2_. The high-energy crystal plane of TiO_2_ is the (101) plane, while the high-energy crystal plane of Fe_2_O_3_ is the (104) plane [48]. Therefore, we constructed the interface model of TiO_2_@Fe_2_O_3_ using the (101) plane of TiO_2_ and the (104) plane of Fe_2_O_3_, and the total number of atoms is 299, as depicted in Figure 10c. Figure 10f is the DOS map of TiO_2_@Fe_2_O_3_, whose spin-up and spin-down show metallicity near the Fermi level, indicating that TiO_2_@Fe_2_O_3_ is a good conductor, and its electronic conductivity is significantly higher than that of anatase TiO_2_ and hematite Fe_2_O_3_. This may be due to the formation of complex chemical bonds at the interface of TiO_2_ and Fe_2_O_3_, where some Ti atoms enter the lattice of Fe_2_O_3_, causing a change in the electron cloud state at the interface. The high electronic conductivity gives an enhancement to the rate performance of TiO_2_@Fe_2_O_3_ microsphere anode material, which is consistent with the experimental results in Figure 8.

## 4. Conclusions

In this study, TiO_2_@Fe_2_O_3_ microsphere anode material is prepared by the homogeneous precipitation method. The results of XRD and Raman spectroscopy indicate that the material consists of anatase TiO_2_ and hematite Fe_2_O_3_ (α-Fe_2_O_3_). SEM and EDS characterization showed that α-Fe_2_O_3_ is uniformly coated on the TiO_2_ surface, and the surface of TiO_2_@Fe_2_O_3_ microspheres is lychee-like, in addition, the monodispersity of the microspheres is good, and the α-Fe_2_O_3_ coating layer accounts for about 7.05% of the total mass. The BET results show that the TiO_2_@Fe_2_O_3_ anode material has a large number of mesopores and micropores on the surface with a specific surface area of 14.72 m^2^/g.

The small amount of Fe_2_O_3_ coating brings a substantial improvement to the electrochemical performance of TiO_2_@Fe_2_O_3_ microsphere anode material. After 200 cycles at a current density of 0.2 C, the specific capacity of TiO_2_@Fe_2_O_3_ anode material increases by 219.3% compared with anatase TiO_2_, reaching 591.5 mAh g^−1^. After 500 cycles at 2 C current density, the discharge-specific capacity of TiO_2_@Fe_2_O_3_ reaches 273.1 mAh g^−1^, which is higher than that of commercial graphite 167.9 mAh g^−1^ under the same test conditions. In addition, TiO_2_@Fe_2_O_3_ microsphere anode material outperforms commercial graphite in terms of cycling consistency as well as rate performance. The EIS results demonstrate that the electronic conductivity of the TiO_2_@Fe_2_O_3_ microsphere anode material is higher than that of anatase TiO_2_ and hematite Fe_2_O_3_, which is the primary reason for its improved rate performance, and it also suggests a synergistic effect between TiO_2_ and Fe_2_O_3_. From DFT calculations, the DOS of TiO_2_@Fe_2_O_3_ shows a metallic nature, revealing the essential reason for the high electronic conductivity of TiO_2_@Fe_2_O_3_. At present, the synergistic effect between TiO_2_ and Fe_2_O_3_ is under further study. This study presents a novel strategy for identifying suitable anode materials for commercial lithium-ion batteries.

## Figures and Tables

**Table 1:** R1 (ohmic resistance) and R2 (charge transfer impedance) values obtained from equivalent circuit fitting for anatase TiO_2_, TiO_2_@Fe_2_O_3_ microspheres, and hematite Fe_2_O_3_ materials in LIBs.

Materials	R1	R2
TiO_2_	2.29	238.35
TiO_2_@Fe_2_O_3_	2.08	119.81
Fe_2_O_3_	4.03	202.17

## Data Availability

Data are available upon request to the corresponding author.

## Supplementary Materials

The following supporting information can be downloaded at: https://www.mdpi.com/article/10.3390/ma16051945/s1, Figure S1: CV curves of hematite Fe_2_O_3_ at the scanning rate of 0.1 mV s^−1^ in the range of 0.01~3.5 V.
