# Supplementary material for: Lychee-like TiO2@Fe2O3 Core-Shell Nanostructures with Improved Lithium Storage Properties as Anode Materials for Lithium-Ion Batteries
Source: Materials (Basel). 2023 Feb 27;16(5):1945. doi: 10.3390/ma16051945 (PMC10004431; doi:10.3390/ma16051945)
Supplement: Supplementary file 1 [file materials-16-01945-s001.zip › materials-2231246-supplementary.pdf]

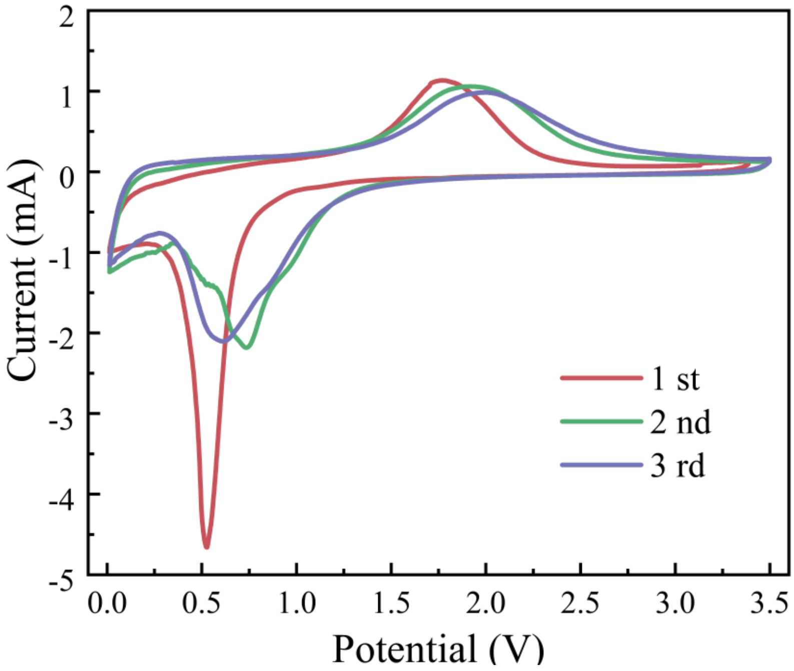

Figure S1. CV curves of hematite Fe_2O_3 at the scanning rate of 0.1 mV s^{-1} in the range of 0.01~3.5 V.

As shown in Figure S1, for the hematite Fe_2O_3 anode material, the reduction peak at 0.5 V corresponds to the multi-step reduction of Fe_2O_3 to Fe^0 according to Equations (S1) and (S2), which shifts to a reduction peak at around 0.7 V during the subsequent cycles, and presumably the disappeared reduction peak corresponds to the insertion of Li^+ into the irreversible site and the generation of the SEI layer. This reduction peak at 0.7 V together with the broad oxidation peak at 1.5~2.5 V during the charging process correspond to the characteristic peaks of the Li^+ insertion/extraction reactions in α-Fe_2O_3 as shown in Equation (S3).

$$Fe_2O_3 + 2Li^+ + 2e^- \rightarrow Li_2(Fe_2O_3) \quad \text{(S1)}$$

$$Li_2(Fe_2O_3) + 4Li^- + 4e^- \rightarrow 2Fe^0 + 3Li_2O \quad \text{(S2)}$$

$$Fe_2O_3 + 6Li \rightarrow 2Fe + 3Li_2O \quad \text{(S3)}$$
